# AGEs and neurodegeneration: the Nrf2/glyoxalase-1 interaction

**DOI:** 10.18632/oncotarget.14232

**Published:** 2016-12-26

**Authors:** Raffaella Mastrocola

**Affiliations:** ^1^ Department of Clinical and Biological Sciences, University of Turin, Turin, Italy

**Keywords:** advanced glycation endproducts, glyoxalase, Nrf2, neurodegenerative diseases

Advanced glycation endproducts (AGEs) are toxic metabolites deriving from reactions between dicarbonyl compounds, such as methylglyoxal, and proteins, which compromise protein function. AGEs effects are counteracted by an enzymatic cycle where glyoxalase (Glo)-1, the limiting enzyme of AGEs detoxification, catalyses the glutathione (GSH)-dependent conversion of methylgyoxal into S-D-lactoylglutathione, which is further metabolised to D-lactate by Glo-2.

A pathogenic role for AGEs in neurodegeneration has been evidenced by several studies showing that the abnormal proteins accumulating in brain in the typical lesions of different diseases are glycated. In addition to physiological ageing, the cerebral accumulation of AGEs can be enhanced by the consumption of saturated fat-rich, sugar-added or high temperature-processed foods contributing to the precocious onset or accelerated progression of neurodegenerative diseases [1,2].

In particular, we recently documented that a high fructose intake evokes AGEs accumulation in hippocampal neurons related to impaired activity of Glo- 1, and this was associated to mitochondrial dysfunction and oxidative stress due to inhibition of nuclear factor-erythroid 2 p45 subunit-related factor 2 (Nrf2). Nrf2 is an essential transcription factor regulating the expression of genes containing an antioxidant-response element (ARE) responsible for protection against oxidative stress and GSH-recycling. Interestingly, in the hippocampus of fructose-fed mice, the inhibition of AGEs production by the administration of pyridoxamine, able to trap the dicarbonyl precursors, also led to restoration of both Glo-1 and Nrf2 activities, limiting the extension of inflammation and oxidative stress [1].

In addition to a healthy diet, low in fat, sugar, and exogenous AGEs, the possibility to enhance Glo- 1 and Nfr2 activities may slow the progression of neurodegeneration. Indeed, studies reporting the positive effects of Nrf2 stimulation on mitochondrial dysfunction and neurodegeneration have been recently reviewed by Esteras and colleagues [3], while the beneficial impact of Glo-1 restoration on specific indicators of Alzheimer’s disease has been evidenced in a transgenic mouse model [4]. In this latter study, the administration of ψ-GSH, a synthetic cofactor of Glo-1 resistant to hydrolysis by intestinal and hepatic γ-GGTs and able to pass the blood brain barrier, reduced cognitive decline, Aβ plaque deposition, protein carbonyl content, and reactive oxygen species. However, these effects were presumably due in part to a general antioxidant effect of GSH replenishing, in addition to specific Glo-1 restoration [4].

Intriguingly, there are increasing indications of a reciprocal influence between Glo-1 and Nrf2: Nrf2 target genes are involved in GSH recycling providing the essential co-factor for Glo-1 enzymatic activity, and v*ice versa*, the depletion of GSH by Glo-1 activity can act as a stimulating factor for Nrf2 activation. However, in condition of excessive AGEs production, which is always accompanied by increased oxidative stress, the concomitant saturation of Glo-1 detoxifying potential and exhaustion of Nrf2 activation might occur.

A previous study also suggests that Nrf2 directly regulates the transcription of Glo-1, since the discovery of a functional ARE in exon 1 of GLO1 gene has been described [5]. In that study, the parallel increase in Glo-1 expression and Nrf2 activation was reported in *in vitro* experiments with specific Nrf2 inducers, such as sulforaphane, and it was demonstrated that this was mediated by interaction of Nrf2 with the regulatory region of GLO1 gene [5]. Similarly, hippocampal and cerebrocortical cultured neurons exposed to high glucose and treated with the natural compound mangiferin, renowned for its antioxidant, anti-inflammatory, and neuroprotective actions, showed upregulation of Glo-1 and enhanced activation of Nrf2. The treatment with the Nrf2-specfic inducer sulforaphane confirmed the Nrf2- dependent upregulation of Glo-1 in the same experimental conditions [6]. In addition, a very recent study has reported the antiparkinsonian effect of vildagliptin in a rat rotenone model that was mediated by the concomitant suppression of AGE-RAGE, suggestive of a possible Glo- 1 involvement, and induction of Nrf2 signalling pathways in the striatus [7].

Therefore, the direct control of Glo-1 expression level by Nrf2 seemed to be contradicted in our study by the increased protein levels of Glo-1, particularly in the dimeric form, detected in the hippocampus of fructose-fed mice, despite Nrf2 nuclear translocation was markedly inhibited [1]. The functional implications of the Glo-1 dimerization and intracellular redistribution, elsewhere described [8], would need to be further investigated to fully understand the mechanisms of regulation of Glo-1 activity and expression. Anyway, it could be postulated that the accumulation of Glo-1 protein content in brain of high fructose mice was due to post-translational modifications that stabilize Glo-1 dimers preventing their degradation, independently of both its enzymatic activity, which was actually reduced, and possibly reduced mRNA expression, that however was not evaluated in our study [1]. This hypothesis is sustained by the observation that the depletion of Glo-1 activity in early and middle stages of Alzheimer’s disease is not associated to a parallel decrease in Glo-1 protein level, which on the contrary is transiently overexpressed, to progressively decline in late stage [4]. In a similar fashion, Nrf2 is temporarily induced by mild oxidative stress conditions, for example in early stage of diabetes, while with excessive reactive oxygen species production the Nrf2-mediated protective mechanism undergoes to exhaustion [6]. These observations may thus indicate a temporal dynamic reciprocal regulation of Nrf2 and Glo-1 during disease development.

In summary, although the interacting mechanisms for Nrf2 and Glo-1 activation should be further cleared in future addressed studies, emerging evidences indicate that the targeting of Nrf2/Glo-1 axis is likely to be a promising adjuvant strategy to limit brain AGEs accumulation and improve neurons antioxidant potential, and thus slow neurodegeneration.
